# Stimuli-Responsible SNARF Derivatives as a Latent Ratiometric Fluorescent Probe

**DOI:** 10.3390/molecules27217181

**Published:** 2022-10-24

**Authors:** Eiji Nakata, Khongorzul Gerelbaatar, Futa Komatsubara, Takashi Morii

**Affiliations:** Institute of Advanced Energy, Kyoto University, Uji 611-0011, Japan

**Keywords:** self-assembled fluorophore, latent fluorescent probe, ratiometry, wash-free, stimuli-responsibility

## Abstract

Fluorescence imaging is a powerful technique for continuous observation of dynamic intracellular processes of living cells. Fluorescent probes bearing a fluorescence switching property associated with a specific recognition or reaction of target biomolecule, that is, stimuli-responsibility, are important for fluorescence imaging. Thus, fluorescent probes continue to be developed to support approaches with different design strategies. When compared with simple intensity-changing fluorescent probes, ratiometric fluorescent probes typically offer the advantage of less sensitivity to errors associated with probe concentration, photobleaching, and environmental effects. For intracellular usage, ratiometric fluorescent probes based on small molecules must be loaded into the cells. Thus, probes having intrinsic fluorescence may obscure a change in intracellular signal if the background fluorescence of the remaining extracellular probes is high. To overcome such disadvantages, it is necessary to minimize the extracellular background fluorescence of fluorescent probes. Here, the design strategy of the latent ratiometric fluorescent probe for wash-free ratiometric imaging using a xanthene dye seminapthorhodafluor (SNARF) as the scaffold of fluorophore is discussed.

## 1. Introduction

A fluorescent probe is a functional molecule whose fluorescent property is changed as a result of stimuli response such as a specific reaction or upon recognition of target molecules. Owing to their high sensitivity, fluorescent probes enable the real-time monitoring of the dynamics of target molecules not only in homogeneously distributed test tubes, but also in heterogeneously distributed environments such as in cells and individual organisms. Thus, fluorescent imaging with a fluorescent probe is indispensable in current biological research [1,2,3,4,5,6].

**Figure 1:**
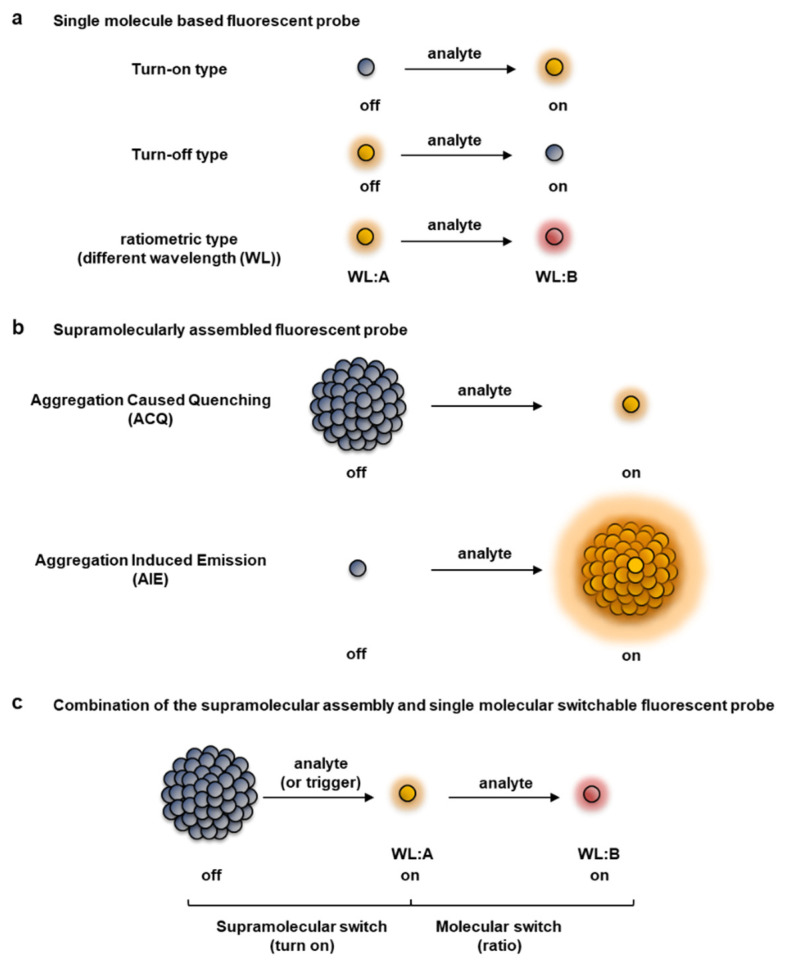
Classification of fluorescent probes based on patterns of the fluorescent response. (**a**) Single molecule type fluorescent probes, including turn-on, turn-off, and ratiometric detection types. (**b**) Supramolecular-type fluorescent probe (turn-on type), which can be roughly classified into aggregation-caused quenching (ACQ) and aggregation-induced emission (AIE) types. (**c**) Combination of a supramolecular assembly-triggered and single molecule switchable fluorescent probe, which is a novel category proposed by us [26,27,28,29,30,31].

Based on the difference in the response in the reaction with target molecules, fluorescent probes are roughly classified into a turn-on or turn-off type, in which the intensity changes from the “off” state to the “on” state, or vice versa, and a ratio type in which the wavelength changes reflecting a colorimetric change (Figure 1a) [7,8,9,10,11,12,13,14,15]. The fluorescent properties of a probe change with a single-molecule-based switch, as well as from a supramolecular interaction between fluorophores (Figure 1b). Some well-known supramolecular processes that contribute to changing the fluorescent properties of a fluorophore are aggregation-caused quenching (ACQ) [16,17,18] and aggregation-induced emission (AIE) [19,20,21,22,23,24]; these mechanisms could be exploited to achieve off–on responses by controlling the self-assembled state, as comprehensively reviewed previously [16,17,18,19,20,21,22,23,24]. Each type of fluorescent probe has its own pros and cons, thus the choice of fluorescent probe depends on the intended use and application [7,8,18,25]. Turn-on and ratio type fluorescent probes are useful from the viewpoint of enabling more sensitive detection of target molecules. The turn-on type of fluorescent probe becomes fluorescent only when a non-fluorescent formed probe reacts with the target molecule. Thus, the target molecule can be detected with high sensitivity. However, it is difficult to quantitatively detect the target molecule based only on its fluorescent intensity, especially in an environment in which the concentration of the fluorescent probe cannot be maintained to be uniform, such as in cells or organisms. Furthermore, probes are susceptible to external factors such as photobleaching that are not related to the reaction of the target molecule.

In the case of a ratiometric fluorescent probe, the intensity changes at different wavelengths occur as a result of the reaction with the target molecule. Therefore, by taking the fluorescence intensity ratio at different wavelengths before and after the reaction, the concentration difference of the fluorescent probe itself can be eliminated and the target molecule can be quantified using an independently measured calibration curve. For intracellular usage with all fluorescent probes, except for genetically coded fluorescent proteins, the fluorophore must be loaded into the cells. Thus, the intrinsic fluorescence of a ratiometric fluorescent probe may obscure a change in the intracellular signal if the background fluorescence of the remaining extracellular probes is high. To reduce background fluorescence, it is necessary to remove the culture medium or wash off excess extracellular probes. Such cumbersome washing processes impose more variation in an assay and are not amenable to high-throughput screening applications. To overcome these disadvantages, a new principle to minimize extracellular background fluorescence, which is classified as a latent ratiometric fluorescent probe, is strongly required [26,27,28].

This study provides a rational design strategy for constructing a ratiometric fluorescent probe and proposes a novel strategy for constructing a latent ratiometric fluorescent probe to overcome the drawbacks described above.

## 2. Design Strategy of a Ratiometric Fluorescent Probe

One of the rational strategies to design a ratiometric fluorescent probe (Figure 1a) is to utilize a ratiometric pH probe [32]. The xanthene dye seminapthorhodafluor (SNARF) is well known as a useful ratiometric fluorescent pH probe [33,34,35]. Thus, the concept and design of a SNARF-based ratiometric fluorescent probe is introduced.

### 2.1. SNARF as the Scaffold of a Ratiometric Fluorescent Probe

**Figure 2:**
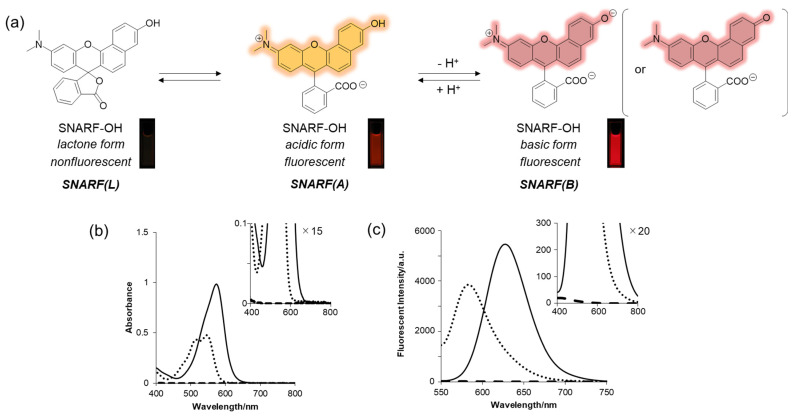
(**a**) Equilibria of SNARF with fluorescent images. (**b**) Absorption and (**c**) emission spectra at different pH values and with different solvents. Solid line: basic form (pH 9.0), dotted line: acidic form (pH 5.0), dashed line: lactone form (in acetonitrile) [SNARF-OH] = 10 μM in 10 mM Tris, Hepes, and acetate buffer (pH 5.0 or 9.0) or acetonitrile [28].

SNARF is a popular ratiometric fluorescent pH probe, which has visible light excitation, dual-emission properties, and a neutral pKa region (Figure 2a) [33]. The phenolic substituent of SNARF is critical for such properties in which an equilibrium exists between the phenol with an emission wavelength of 583 nm (excitation maximum: 544 nm) (SNARF(A)) and the phenolate anion with an emission wavelength of 627 nm (excitation maximum: 573 nm) (SNARF(B)) (Figure 2) [25,26,27,28,29,30,31,32,33]. Notably, one of the most favorable optical properties of SNARF is the good balance of SNARF(A) and SNARF(B), making it suitable as a ratiometric pH probe with a dual-emissive property and excitation wavelength [33]. Further equilibria involving the formation of colorless and non-fluorescent lactone (SNARF(L)) occur in anhydrous conditions such as in the presence of acetonitrile [27,28]. Controlling these equilibria on demand is the basis of this rational strategy to construct a SNARF-based ratiometric fluorescent probe [26,27,28,29,30,31].

**Figure 3:**
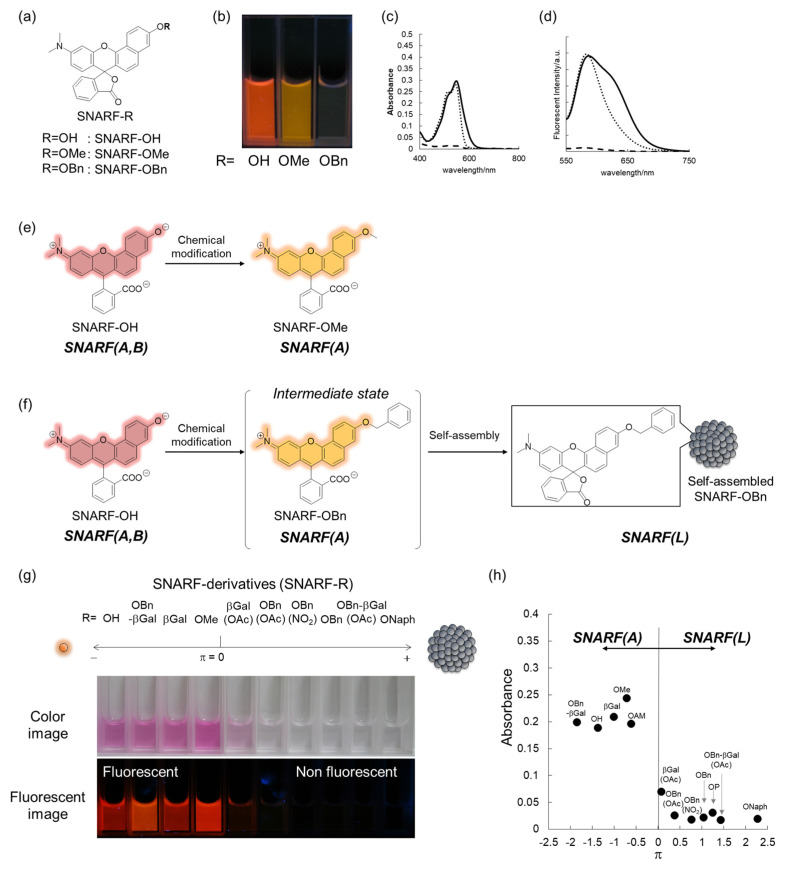
(**a**–**d**) Structure (**a**), photograph at pH 7.0 (**b**), absorption spectra (**c**), and emission spectra (**d**) (excitation at 534 nm) of SNARF-OH (solid lines), SNARF-OMe (dotted lines), and SNARF-OBn (dashed lines). Schematic illustration of (**e**) SNARF-OMe to form SNARF(A) and (**f**) SNARF-OBn to form SNARF(L) in aqueous media. (**g**) Comparison of the absorption and fluorescence of SNARF derivatives divided by the Hansch–Fujita hydrophobic parameter (π) of the inserted substituent of SNARF. (**h**) Relationships between the π value of the inserted substituent and the absorbance measured in aqueous media. See [28] for further details.

To rationally control these equilibria under aqueous media, various SNARF derivatives, in which the phenolic substituent was protected, were prepared and the structure–activity relationships were evaluated [28]. As typical examples, SNARF-OMe and SNARF-OBn, in which the phenolic group of SNARF was converted to methoxy and benzoxy substituents, respectively, are shown in Figure 3a–d. Both SNARF-OMe and SNARF-OBn showed different fluorescent properties compared with SNARF-OH. Usually, SNARF derivatives with a protected phenolic group exhibit the spectral characteristics of the acidic form with pH independency (Figure 3e) [26]. In contrast, SNARF-OBn existed as SNARF(L) in aqueous media through the formation of self-assembled clusters that themselves created a hydrophobic environment [28]. Therefore, the clustered state displayed no significant fluorescence emission in the lactone form (SNARF(L); Figure 3f), which was defined as the self-assembly induced lactone formation (SAILac) [28]. The assembly capabilities of the assessed SNARF derivatives could be rationalized by the hydrophobicity of the protective group of SNARF, which is the Hansch–Fujita hydrophobic parameter (π) [36,37,38], as shown in Figure 3g,h. By controlling these different properties, two classes of ratiometric fluorescent probes could be designed: a classical ratiometric fluorescent probe using the equilibria between SNARF(A) and SNARF(B) and a latent ratiometric fluorescent probe defined as having a two-step switch (turn-on and then a ratiometric switch) using these three forms (SNARF(A), SNARF(B), and SNARF(L)).

### 2.2. Classical Design of a Ratiometric Fluorescent Probe Based on a SNARF Scaffold

**Figure 4:**
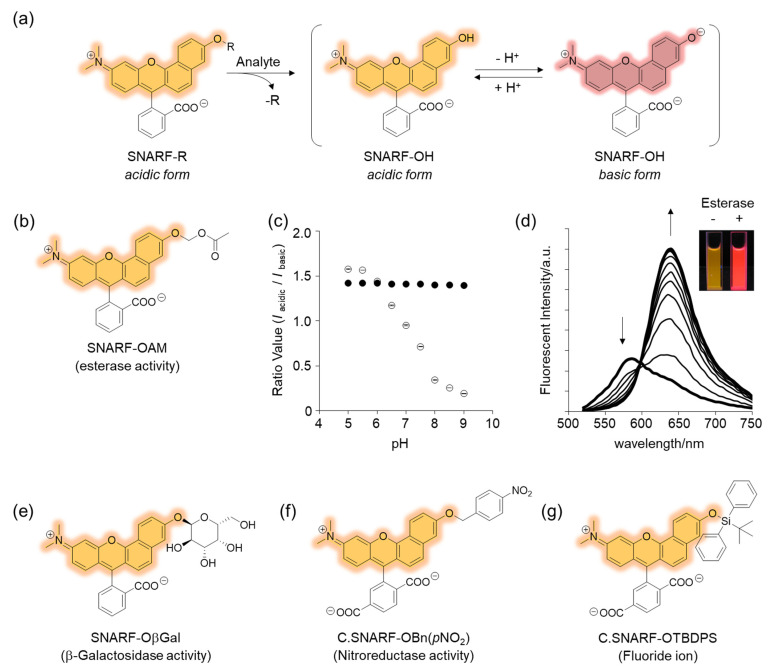
Rationally designed SNARF-based ratiometric fluorescent probe. (**a**) Schematic illustration of SNARF-R-based classical ratiometric fluorescent probe. (**b**) Structure of SNARF-OAM. (**c**) The ratio of fluorescence intensities of the acidic and basic forms plotted versus pH (open circles: SNARF-OH, filled circles: SNARF-OAM). (**d**) Fluorescence spectral change of SNARF-OAM after adding porcine liver esterase. Inset: fluorescent photograph of SNARF-OAM with or without esterase treatment. See [29] for further details. (**e**–**g**) Various types of SNARF-based ratiometric fluorescent probes: (**e**) SNARF-OβGal for β-galactosidase activity, (**f**) C. SNARF-OBn(*p*NO_2_) for nitroreductase activity, and (**g**) C. SNARF-OTBDPS for fluoride ion [30].

The phenolic substituent-protected SNARF exhibited spectral characteristics of the acidic form with pH independency (Figure 3e). Following removal of the protecting group, either chemically or biologically, the resulting SNARF (SNARF-OH) existed in an equilibrium of an acidic and basic form, the ratio of which depended on the pH. This is the classical mechanism of a ratiometric fluorescent probe, including a SNARF scaffold, as shown in Figure 4a. For example, SNARF derivatives in which the phenolic moiety is masked by an acetoxymethyl group (SNARF-OAM) could serve as a ratiometric fluorescent probe for esterase (Figure 4b) [27,29]. SNARF-OAM exists in an acidic form and the fluorescent ratio (*R* = *I*_acidic_/*I*_basic_) was constant from pH 5.0 to 9.0. In contrast, the fluorescent ratio of SNARF drastically changed from the acidic form to basic form and this shift was ascribed to the equilibria of SNARF-OH (pKa = 7.6). Thus, the changing ratio due to the conversion of SNARF-OAM to SNARF-OH would be readily observable at pH conditions above the neutral region (Figure 4a). Enzymatic conversion of SNARF-OAM by porcine liver esterase to SNARF-OH could be monitored by the ratiometric change in the emission spectra, and a change in color could be observed (Figure 4d) [29]. Notably, several types of ratiometric fluorescent probes could be designed using the same strategy, as illustrated in Figure 4e–g [30].

However, one of the major drawbacks of this ratiometric fluorescent probe is the intrinsic fluorescence of SNARF-OAM that causes strong extracellular fluorescence, posing a challenge for intracellular monitoring without a washing procedure [27,28]. This limitation could be overcome through the design of a latent ratiometric fluorescent probe.

### 2.3. Design Principle of a Self-Assembled Fluorophore Based on a SNARF Scaffold as a Latent Ratiometric Fluorescent Probe

**Figure 5:**
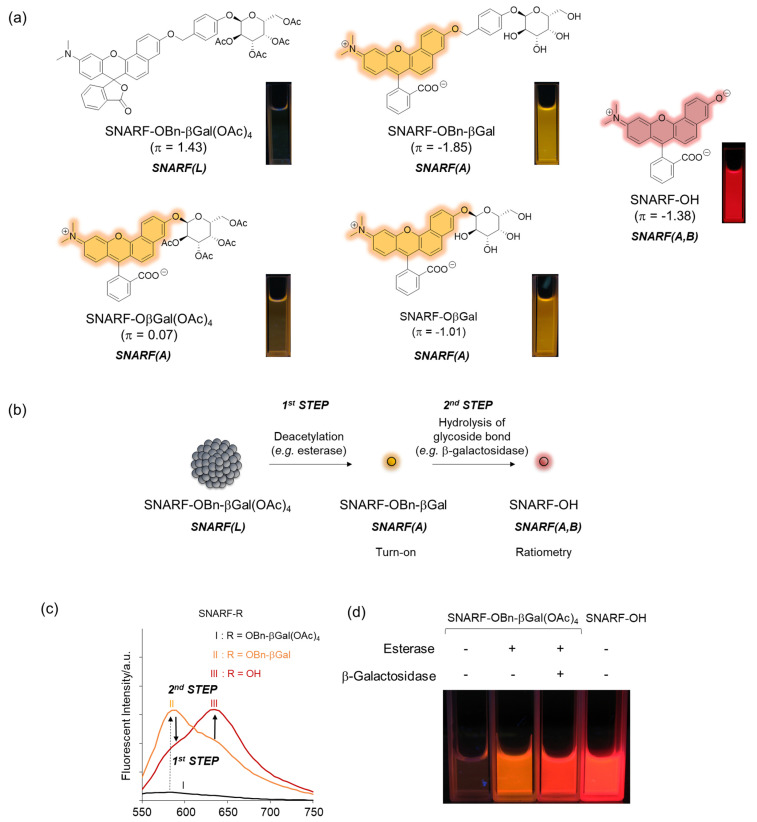
Latent ratiometric fluorescent probe based on the self-assembled lactone form of SNARF. (**a**) Structure of SNARF-OBn-βGal(OAc)_4_, SNARF-OBn-βGal, and SNARF-OH. As the control compound, the structures of SNARF-OβGal(OAc)_4_ and SNARF-OβGal are shown. (**b**) Schematic illustration of a SNARF-OBn-βGal(OAc)_4_-based latent ratiometric fluorescent probe responsive to esterase and β-galactosidase [28]. (**c**) Comparison of fluorescent spectra of SNARF-OBn-βGal(OAc)_4_, SNARF-OBn-βGal, and SNARF-OH. (**d**) Fluorescent image of SNARF-OBn-βGal(OAc)_4_ treated by esterase and/or β-galactosidase.

As described in Section 2.1, the equilibria between the assembled and dispersed state of the phenolic substituent-protected SNARF could be classified by the π value of R; according to our previous research, at π < 0, SNARF(A) is formed and, at π > 0, SNARF(L) is formed [28]. Based on this theory, a two-step activatable fluorescent probe could be rationally designed [28]. As an example, the π value of SNARF-OBn-βGal(OAc)_4_ is 1.43 and that of deacetylated SNARF-OBn-βGal is −1.85; therefore, SNARF-OBn-βGal(OAc)_4_ in SNARF(L) form and SNARF-OBn-βGal in SNARF(A) form were designed, as shown in Figure 5 [27]. SNARF-OBn-βGal(OAc)_4_ showed very weak fluorescence and SNARF-OBn-βGal showed strong fluorescence identified with SNARF(A) (Figure 5a). Therefore, the deacetylation reaction of SNARF-OBn-βGal(OAc)_4_ produced SNARF-OBn-βGal, indicating that the turn-on fluorescence change occurred. Subsequently, SNARF-OH was produced from SNARF-OBn-βGal through hydrolysis of the glycoside bond, allowing for the ratiometric change in fluorescence to be observed (Figure 5b,c). As shown in Figure 5d, although SNARF-OBn-βGal(OAc)_4_ showed weak fluorescence, strong fluorescence appeared in the presence of esterase and a fluorescence color change was observed in the presence of both esterase and β-galactosidase. The fluorescent color of the resultant product was the same with SNARF-OH. Notably, SNARF-OβGal(OAc)_4_, which also has esterase and galactosidase-activatable properties, existed in SNARF(A) form in the aqueous condition because a self-assembled cluster could not be formed because of the lower hydrophobicity of the phenolic substituent (π = 0.07). These results strongly indicated that a latent ratiometric fluorescent probe could be designed rationally.

## 3. Advantage of a Latent Ratiometric Fluorescent Probe for Intracellular Monitoring

**Figure 6:**
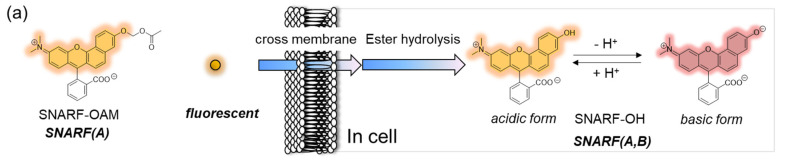

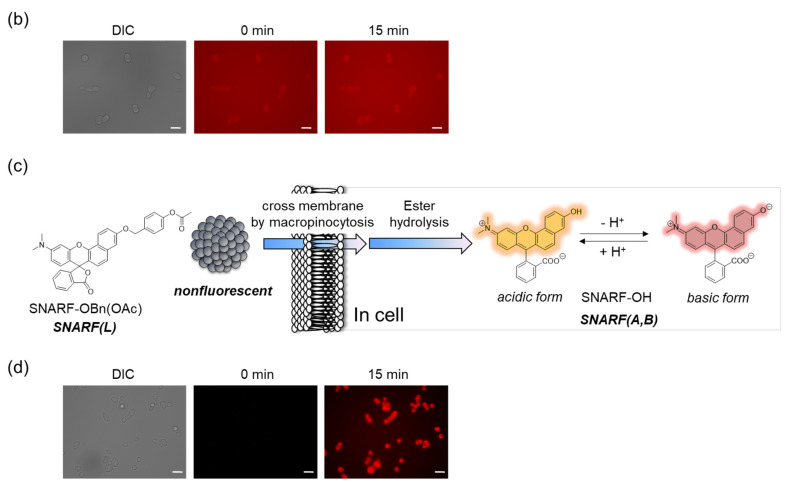
Comparison of the classical ratiometric fluorescent probe and latent ratiometric fluorescent probe. (**a**) Schematic illustration of SNARF-OAM as the intracellular ratiometric fluorescent pH probe activated by ester hydrolysis. (**b**) Fluorescent images of V79 cells in FBS-containing EMEM medium (scale bar: 30 μm). (**c**) Schematic illustration of SNARF-OBn(OAc) as the latent ratiometric fluorescent pH probe activated by ester hydrolysis. (**d**) Fluorescent images of V79 cells in FBS-containing EMEM medium (scale bar: 30 μm). See [27] for further details.

**Figure 7:**
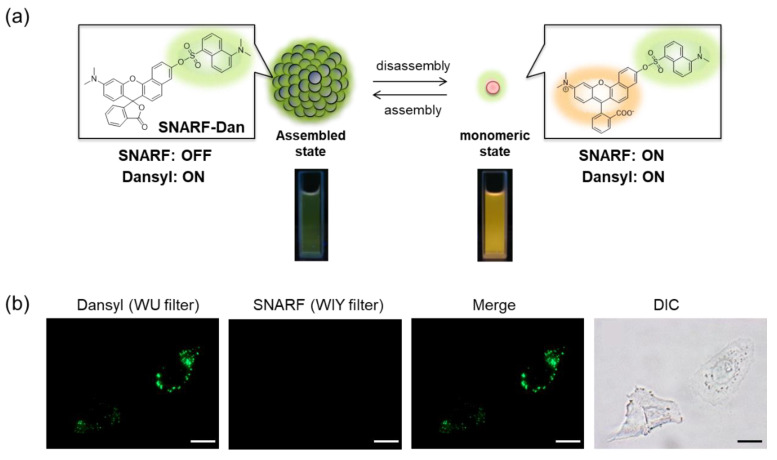
(**a**) Schematic illustration of colorimetric fluorescent change of SNARF-Dan between assembled and disassembled states. (**b**) Fluorescent images and bright-field transmission of HeLa cells pre-treated with SNARF-Dan in FBS-containing EMEM medium (scale bar: 10 μm). See [28] for further details.

SNARF is mostly used as a ratiometric fluorescent pH probe [25,32]. In this section, we focus on applying SNARF as a ratiometric pH probe to monitor the intracellular pH and overcome the current challenges described above [26,27,28]. SNARF itself has low cell permeability; therefore, the phenolic group of SNARF should be converted to esterase substrates such as acetate (OAc) and acetoxymethyl ester (OAM) to increase the cell permeability (Figure 6a) [32]. However, the intrinsic fluorescence of SNARF-OAM (π = −0.62) causes strong extracellular fluorescence, as shown in Figure 6b, which makes it difficult to distinguish between the inside and outside of the cell without a washing procedure. Thus, a latent ratiometric fluorescent probe that responds to esterase and produces the pH-sensitive fluorescence of SNARF can overcome these difficulties. To achieve this, SNARF-OBn(OAc) (π = 0.90) was designed as a latent ratiometric fluorescent pH probe activated by esterase (Figure 6c) [27]. SNARF-OBn(OAc) displayed negligible absorption and fluorescence emission because of the formation of self-assembled clusters approximately 70 nm in size. Moreover, esterase-treated SNARF-OBn(OAc) showed fluorescence emission characterized as SNARF-OH with the diffusion of the self-assembled clusters [28]. It should be noted that SNARF-OBn(OAc) also formed self-assembled clusters in the cell culture media such as EMEM medium with FBS [27]. Upon addition of SNARF-OBn(OAc), the medium was scarcely fluorescent and only the uptake of the fluorescent intensity inside the cell was observed after long-term incubation (about 15 min incubation) (Figure 6d). The intracellular pH values could be measured not only for adherent cells [27], but also for the loosely adherent or floating cells [28] using SNARF-OBn(OAc) because of the advantage of wash-free procedure. These results established the superiority of SNARF-OBn(OAc) as an intracellular pH probe with notable cell permeability and low background noise. These properties should be advantageous for in vivo applications because of the difficulty in washing out the redundant probe. Moreover, a latent ratiometric fluorescent pH probe can be designed to target different activators by changing the substituent for the phenolic-protecting group of SNARF-OH based on our proposed strategy [28]. SNARF-OBn(*p*NO_2_) was designed as a latent fluorescent pH probe activated in hypoxic conditions [26]. The mechanism of cellular uptake of self-assembled SNARF derivatives was investigated using SNARF-Dan (π = 1.81), which could monitor the self-assembled state by colorimetric change in the fluorescence of Dansyl and SNARF fluorophores (Figure 7) [28]. That is, the self-assembled cluster of SNARF-Dan showed strong Dansyl fluorescence without SNARF fluorescence, but disassembled SNARF-Dan has the fluorescence of both Dansyl and SNARF (Figure 7a). When SNARF-Dan was incubated with HeLa cells, endosome-like punctate fluorescence derived from Dansyl fluorophore was observed inside the cells for a long time period (Figure 7b). Following a detailed assessment of the process of cellular uptake of self-assembled SNARF-Dan, it was suggested that the self-assembled cluster of SNARF derivatives was introduced into the cell via macropinocytosis. Though the details of how endosomal escape after macropinocytic uptake of self-assembled SNARF derivatives occurs still remain ambiguous, the utility of self-assembled SNARF derivatives is strongly indicated [28]. 

## 4. Conclusions

This opinion has summarized the construction methodologies for a ratiometric fluorescent probe based on a SNARF scaffold. Two types of ratiometric fluorescent probes could be rationally designed based on our strategy. The choice of the most appropriate probe depends on the intended use and application, as each form has its own advantages and disadvantages. The advantages of a latent fluorescent ratiometric probe include effective intracellular uptake and reduction of background noise. Therefore, the proposed rational strategy for designing a latent ratiometric fluorescent probe has great potential for tissue or in vivo applications as a targeted cell-specific activatable ratiometric fluorescent probe.

## Data Availability

Not applicable.
